# Health promoting schools and children’s oral health related quality of life

**DOI:** 10.1186/1477-7525-11-205

**Published:** 2013-12-10

**Authors:** Zamros YM Yusof, Nasruddin Jaafar

**Affiliations:** 1 Department of Community Oral Health and Clinical Prevention, Faculty of Dentistry, University of Malaya, 50603 Kuala Lumpur, Malaysia; 2 Community Oral Health Research Group, University of Malaya, 50603 Kuala Lumpur, Malaysia

**Keywords:** Evaluation, Health promoting school, Oral health promotion, Oral health related quality of life, Schoolchildren

## Abstract

**Background:**

The study objective was to compare children’s oral health related quality of life (OHRQoL) in schools with 6 years of implementation of a health promoting school model in Malaysia, i.e. the *Doktor Muda* Programme (DMP) and in schools without the DMP.

**Methods:**

This report was part of a larger study to evaluate the DMP impact on schoolchildren’s oral health knowledge, attitudes, behaviour, caries progression and OHRQoL. It was conducted in Negri Sembilan state. The sample comprised 3455, Year 6 (11–12 year old) children; 1282 from DMP (intervention) and 2173 from non-DMP (control) schools. The Malay Child-OIDP index was used to evaluate children’s levels of oral impacts on 8 daily performances after 6 years of DMP implementation (2006–2011). Prevalence, score, impact intensity, causes and extent of impacts were compared. Chi-square and Mann–Whitney tests were used in the data analysis.

**Results:**

Overall response rate was 95.1%. Prevalence of overall impacts was 57.8% and 60.8% (mean total impact score was 7.10 and 7.77) in the intervention and control group, respectively. The three most frequently affected performances in both groups were eating, cleaning teeth and emotional stability. Significantly less DMP children had oral impact on cleaning teeth (*p* = 0.034). The majority of children with impacts in both groups reported ‘very little’ to ‘moderate’ levels of impact intensity. Significantly more DMP children reported having ‘very little’ and ‘little’ levels of impact intensity on cleaning teeth (*p* = 0.037) and emotional stability (*p* = 0.020), respectively. Significantly less DMP children reported having ‘very severe’ level of impact intensity on speaking (*p* = 0.038). The most prevalent cause of impacts in both groups was toothache. Significantly less DMP children reported bleeding gums (*p* = 0.016) and presence of plaque/calculus as causes of impacts (*p* = 0.032). About 75% of children with impacts in both groups reported having up to four daily performances affected.

**Conclusion:**

This study showed that the health promoting school model, i.e. the *Doktor Muda* Programme for primary schools in Malaysia had some positive impacts on 11–12 year old children’s oral health related quality of life.

## Background

Health promoting school is defined as ‘a school constantly strengthening its capacity as a healthy setting for living, learning and working’ [1]. The primary aim is to increase the school’s capacity in promoting the health of school population, families and the community as they do learning [2]. Health promoting schools have been shown to be effective in improving children’s oral health outcomes. In Brazil, health promoting schools were associated with significant increase in children’s caries-free teeth and reduction in the number of teeth injuries [3]. In Canada, health promoting schools were effective in reducing children’s caries prevalence and those requiring urgent dental treatment [4]. Health promoting schools that incorporated oral health education and promotion programmes were also effective against caries, plaque and gingival bleeding scores in children. Significant improvement in the intermediate oral health outcomes, i.e. self-reported tooth brushing frequency, use of topical fluorides, flossing, intake of sweet food and drinks and between-meal snacking were also reported [5-8]. However, its effect on children’s oral health related quality of life (OHRQoL) has not been investigated. The closest related study in children was reported in Brazil that evaluated the relationship between oral health education delivered in community primary care clinic and adolescents’ OHRQoL [9].

OHRQoL instruments have been developed to measure subjective oral impacts on daily performances and thus, quality of life [10]. Their use enabled important information on the functional and social dimensions of dental diseases and illness to be collected. If the oral impacts on daily performances were severe, their effects on quality of life would also be severe. In the present study, the Child Oral Impacts on Daily Performances (Child-OIDP) index had been chosen as the instrument to evaluate children’s levels of OHRQoL [11]. The index was developed in Thailand in 2004 among 11–12 year old schoolchildren [11]. The Child-OIDP derived its theoretical framework based on modifications from the WHO’s International Classification of Impairment, Disabilities and Handicaps [10,12]. Thus, it allowed measurement of a range of OHRQoL dimensions including oral health impairments, functional limitations and disability [13-15], making it suitable to measure oral impacts in children. The Child-OIDP index measured oral impacts on eight performances, i.e. eating, talking, cleaning teeth, relaxing, emotional stability, smiling, doing schoolwork and socialising. It is relatively short, comprehensive and easy to use. Its scoring system directly relates the frequency and severity of impacts with the impact scores. In the present study, a self-administered Malay Child-OIDP index was used [16].

In Malaysia, the health promoting school model used was a child-to-child health promotion approach called the *Doktor Muda* (Junior Doctors) Programme (DMP). It had been introduced in primary schools since the late 1980s [17]. In 2004, the number of DMP schools was 347. The number increased to 1255 in 2010 with 33,440 trained *doktor muda* (DM) all over Malaysia [18]. However the effect of the DMP on health or oral health has not been systematically evaluated.

The objective of the study was to compare children’s OHRQoL in schools with 6 years of implementation of DMP and in schools without the DMP. The hypothesis tested was that children from DMP schools (intervention group) have significantly better OHRQoL compared to children from non-DMP schools (control group).

## Methods

This study was part of a larger retrospective cohort study to evaluate the DMP effect on children’s levels of oral health knowledge, attitudes, behaviour, caries progression and OHRQoL after 6 years of DMP implementation (2006–2011 enrolment). The study was conducted in the state of Negri Sembilan, Malaysia. This state has 347 primary schools distributed over 7 districts with a total enrolment of 112,202 students in 2011 [19]. Children start primary school at 6–7 years old (Year 1) until 11–12 years old (Year 6). Negri Sembilan was chosen because apart from the population’s broad socio-economic status, it has successfully implemented the DMP in the most systematic way under the State Health Department’s guidance. One of the schools had won ‘National DMP School Excellence Award’ in 2008 and one student won the ‘National *Doktor Muda* Icon’ award in the same year. This recognition led to a visit by a WHO representative to the state winning school in 2009 [20].

### Sample selection

Sample size for the study was based on the assumption that the DMP would reduce children’s mean caries increment by about 12% over 6 years (2006–2011) as compared to the control group. This yielded the largest sample size to include all the research variables in the main study. Using a Power and Sample Size Calculations Programme version 3.0.14 [21] and based on the above assumption, sample size for the intervention (children in DMP schools) and control group (children without DMP), with 80% power and 5% Type 1 error, was 1100. The sample in each group was increased by 20% to 1320 to compensate for dropouts [22]. According to Negri Sembilan State Health Department Health Promotion Unit, the number of DMP schools in 2011 was 71 [23]. Of these, 16 schools, with 1340 Year 6 students fulfilled the study criteria, i.e. had implemented the DMP inclusive of 2006–2011. Thus, all the 1340 students were included in the intervention group. For control, each DMP school in the intervention group was matched with a non-DMP school, by nearest location in the neighbourhood to ensure that both groups had similar sociodemographic characteristics. Once the control school was selected, all Year 6 students who fulfilled the study criteria were included. If the number of Year 6 students in the intervention school was higher than the number of Year 6 students in the control school, the next nearest non-DMP school was included as control. To overcome the probable cluster effect in selecting the control, sample size for the control group was multiplied by a factor of 1.8, n = 1100 × 1.8, and increased by 20% to 2376 [22]. Thus, based on this initial calculation, the sample comprised 30 control schools with 2394 Year 6 students to match 16 intervention schools with 1340 students.

The DMP effect on children’s OHRQoL after six years was assessed by distributing the Malay Child-OIDP index to Year 6 (11–12 year old) students in the intervention and control group in 2011. The inclusion criteria for the intervention group were students in Year 6 who started school from Year 1 (in 2006) and remained until Year 6 (2011) in DMP national school which had run the programme continuously for at least 6 years inclusive of 2006–2011. The inclusion criteria for the control group were students in Year 6 (in 2011) with no exposure to DMP since Year 1 (2006) in non-DMP school. It was assumed that the level of OHRQoL among 6–7 year olds (Year 1) in 2006 between the intervention and control group was not statistically significant. The exclusion criteria were children with genetic skeletal or dental anomalies, chronic medical history or who were on long term medication which affected the teeth development.

### Conduct of study

Ethical approval for the study was granted by Medical Ethics Committee, Faculty of Dentistry, University of Malaya. Permission to conduct the study was obtained from the Ministries of Health and Education, Negri Sembilan State Health and Education Departments, respective schools and parents of schoolchildren. Data collection took six months from July-November 2011. On the first visit, all Year 6 students were screened for the inclusion and exclusion criteria. Those who fulfilled the study criteria were given a parent’s form which contained parent’s demographic information and consent for the study. On the second visit, all students with parent’s consent were given the Malay Child-OIDP index to answer. They also answered a second set of validated questionnaire on oral health knowledge, attitudes and reported oral health behaviour. Caries data for 2006–2011 were obtained from the respective district dental clinics. These retrospective caries data were obtained from annual dental examination clinical records carried out by the school dental service. The school dental service provided examination and treatment to all schoolchildren. All treatment providers were calibrated annually to ensure consistency in diagnosis and treatment planning. No radiographs were taken and decay was recorded at the level of cavitation using a mouth mirror and a WHO/Community Periodontal Index of Treatment Need (CPITN) probe [24].

### *Doktor Muda* Programme (DMP)

This is a child-to-child school-based health promotion programme where a selected group of schoolchildren, known as DM, are trained and empowered to give health education to their peers and conduct health activities at school [17]. They act as a role model for healthy behaviours and lifestyles. The selection and training of 25–30 DM (Year 4 – Year 6) per school, was conducted by teacher trainers who were trained by health education officers from the State Health Department. The teachers acted as moderators and supervisors for the DM to avoid inappropriate pressures such as bullying by the DM over their peers.

At school, DM are trained through a modular curriculum in a range of health topics based on ten themes including personal hygiene, environmental hygiene, oral health, mental health, prevention of infectious disease, safety and injury prevention, healthy nutrition and diet, healthy lifestyles, and healthy teenagers. Once trained, DM will deliver health education to peers throughout school year. Routine activities include a 10-minute health talk by DM during school assembly, a once-a-week short health talk in ‘adopted classrooms’ before lesson begins, distributing health leaflets to peers, preparing scrap books with health messages, putting up health posters, supervising hand washing exercise, supervising weekly toothbrushing with fluoride toothpaste and conducting DM Club activities on co-curricular days [17].

In addition, through DMP initiatives, DM monitor their peers for healthy school environment and prevention of endemic diseases, i.e. dengue and malaria from occurring. They keep a record of health problems, e.g. cases of vomiting, diarrhoea, and flu-like symptoms involving schoolchildren, and involve in school health activities, i.e. campaigns on anti-smoking and drug abuse. On school health day, DM help teachers to record student’s health report card for weight, height, eye test, health inspection and personal hygiene. Other activities include organising health quiz, drawing, essay writing, and public speaking competitions. DM are also trained to treat minor injuries through first aid. Outside school activities include visits to the local clinic, hospital, community health centre and undertaking health campaigns at community elderly centres. A national DMP convention is held every 2 years to allow for exchanges of ideas and promote best practices among the schools. DM exhibitions and competitions on various health promotion initiatives are also carried out.

### Data analysis

Data were analysed using SPSS 17.0. Each performance of the Malay Child-OIDP index was calculated by multiplying the frequency (0 to 3) and severity (0 to 3) of impact. The scores of the eight performances were summed up. Overall score was the sum divided by 72 (maximum possible score) and multiplied with 100 to give a percentage score. As a result, a child can have no oral impact (score = 0) or maximum oral impacts (score = 100) on his eight daily performances.

In addition to prevalence and impact score, the impact intensity and extent of impacts were also reported and compared between the intervention and control group. For intensity of impact, each performance score was categorised into ‘no impact’ (score = 0), ‘very little’ (score = 1), ‘little’ (score = 2), ‘moderate’ (score = 3-4), ‘severe’ (score = 6) and ‘very severe’ (score = 9) levels of impact intensity [25]. For extent of impacts, the number of OIDP performances affected by oral conditions was compared between groups.

The children’s OIDP scores were non-normally distributed. Thus, between group score differences were analysed using Mann–Whitney test. Differences in proportions between categorical data were assessed using Chi-square test. Weighted data were used to account for clustering effect of urban/rural distribution of the sample. In all analyses, level of significance was set at *p* < 0.05.

## Results

Overall, 1282 and 2173 children in the intervention and control group fulfilled the study criteria, respectively. The overall response rate was 95.1% (intervention = 95.5%, control = 94.8%). There were no significant socio-economic differences between the intervention and control group in terms of father’s and mother’s education levels, income levels and distribution of children in all the seven districts. However, significantly higher proportions of control children were female (54.5%, *p* = 0.012) and came from rural schools (59.5%, *p* = 0.045). As a result, subsequent data analyses were based on the weighted data to account for differences in the urban/rural distribution of the children. Both the intervention and control group comprised predominantly of Malay children, 96.5% and 91.1%, respectively (Table 1).

**Table 1:** Demographic characteristics of the sample by group (N = 3285)

**Variable**	**Overall**	**Intervention**	**Control**	***p*-value†**
	**N (%)**	**n (%)**	**n (%)**	
Gender				
Male	1549 (47.2)	612 (50.0)	937 (45.5)	0.012*
Female	1736 (52.8)	612 (50.0)	1124 (54.5)	
Race				
Malay	3059 (93.1)	1181 (96.5)	1878 (91.1)	
Chinese	23 (0.7)	2 (0.2)	21 (1.0)	
Indian	148 (4.5)	34 (2.8)	114 (5.5)	<0.001*
Other^1^	55 (1.7)	7 (0.6)	48 (2.3)	
District				
Seremban	1240 (37.7)	448 (36.6)	792 (38.4)	
Kuala Pilah	456 (13.9)	166 (13.6)	290 (14.1)	
Port Dickson	465 (14.2)	178 (14.5)	287 (13.9)	
Rembau	347 (10.6)	125 (10.2)	222 (10.8)	0.487
Jelebu	247 (7.5)	102 (8.3)	145 (7.0)	
Tampin	169 (5.1)	58 (4.7)	111 (5.4)	
Jempol	361 (5.1)	147 (12.0)	214 (10.4)	
Location of school				
Urban	1373 (41.8)	539 (44.0)	834 (40.5)	0.045*
Rural	1912 (58.2)	685 (56.0)	1227 (59.5)	
Father’s education level^2^				
Primary school	336 (11.9)	115 (11.0)	221 (12.5)	
Secondary school	1776 (63.1)	671 (64.3)	1105 (62.3)	0.291
College	290 (10.3)	115 (11.0)	175 (9.9)	
University	414 (14.7)	142 (13.6)	272 (15.3)	
Mother’s education level^2^				
Primary school	365 (12.7)	129 (12.2)	236 (13.1)	
Secondary school	1833 (64.0)	692 (65.4)	1141 (63.2)	0.688
College	346 (12.1)	125 (11.8)	221 (12.2)	
University	319 (11.1)	112 (10.6)	207 (11.5)	
Family income^2^				
< RM400	165 (5.7)	56 (5.2)	109 (6.0)	
RM400-749	586 (20.3)	196 (18.3)	390 (21.5)	
RM750-2299	1226 (42.5)	479 (44.6)	747 (41.2)	0.063
RM2300-5599	618 (21.4)	244 (22.7)	374 (20.6)	
>RM5600	290 (10.1)	98 (9.1)	192 (10.6)	

†Chi-square test; ^1^Other consists of 49 Orang Asli (indigenous people), 4 Sikh, 1 Filipino and 1 Pakistani; ^2^Sample did not equal to N = 3285 due to missing data.

*Level of significant *p* < 0.05.

Table 2 shows the prevalence and score of OIDP between the intervention and control group. After six years of DMP, overall, almost 60% of children reported at least one oral impact in their daily performances in the last three months. Overall, the affected performances from the highest to the lowest prevalence were on eating (40.2%), cleaning teeth (31.3%), emotional stability (24.1%), smiling (22.5%), speaking (19.3%), relaxing (19.2%), socialising (18.8%), and studying (14.5%). Similar trend was also observed for the intervention and control group, respectively.

**Table 2:** Weighted OIDP prevalence and performance score for the 8 items of the Child-OIDP scale (n = 3272)

		**Daily performance**							
**Oral impacts**	**Overall (n = 1971)**	**Eating (n = 1329)**	**Speaking (n = 642)**	**Cleaning (n = 1037)**	**Relaxing (n = 640)**	**Emotion (n = 799)**	**Smiling (n = 745)**	**Studying (n = 487)**	**Socialising (n = 630)**
Prevalence % (all)	59.7	40.2	19.3	31.3	19.2	24.1	22.5	14.5	18.8
Impact score (all)									
Range^1^	0 – 100	0 – 9	0 – 9	0 – 9	0 – 9	0 – 9	0 – 9	0 – 9	0 – 9
Mean (SD)	7.52 (11.7)	1.04 (1.7)	0.51 (1.3)	0.91 (1.8)	0.55 (1.4)	0.74 (1.7)	0.67 (1.6)	0.42 (1.3)	0.57 (1.5)
Percentiles^2^	(0, 2.8, 9.9)	(0, 0, 2)	(0, 0, 0)	(0, 0, 1)	(0, 0, 0)	(0, 0, 0)	(0, 0, 0)	(0, 0, 0)	(0, 0, 0)
	Overall (n = 717)	Eating (n = 493)	Speaking (n = 229)	Cleaning (n = 360)	Relaxing (n = 228)	Emotion (n = 289)	Smiling (n = 268)	Studying (n = 171)	Socialising (n = 226)
**Intervention (n = 1222)**									
Prevalence (%)	57.8	39.6	18.5	*^a^28.8	18.2	23.4	21.5	13.5	18.1
Impact score									
Range^1^	0 – 100	0 – 9	0 – 9	0 – 9	0 – 9	0 – 9	0 – 9	0 – 9	0 – 9
Mean (SD)	7.10 (11.1)	1.03 (1.6)	0.49 (1.2)	0.85 (1.7)	0.53 (1.4)	0.69 (1.6)	0.63 (1.5)	0.37 (1.1)	0.54 (1.4)
Percentiles^2^	(0, 2.8, 9.7)	(0, 0, 2)	(0, 0, 0)	(0, 0, 1)	(0, 0, 0)	(0, 0, 0)	(0, 0, 0)	(0, 0, 0)	(0, 0, 0)
	Overall (n = 1254)	Eating (n = 836)	Speaking (n = 413)	Cleaning (n = 677)	Relaxing (n = 412)	Emotion (n = 510)	Smiling (n = 477)	Studying (n = 316)	Socialising (n = 404)
**Control (n = 2050)**									
Prevalence (%)	60.8	40.5	19.8	^a^32.8	19.8	24.5	23.1	15.0	19.3
Impact score									
Range^1^	0 – 100	0 – 9	0 – 9	0 – 9	0 – 9	0 – 9	0 – 9	0 – 9	0 – 9
Mean (SD)	7.77 (11.9)	1.06 (1.7)	0.52 (1.4)	0.95 (1.8)	0.56 (1.4)	0.76 (1.7)	0.70 (1.6)	0.45 (1.4)	0.59 (1.5)
Percentiles^2^	(0, 2.8, 11.1)	(0, 0, 2)	(0, 0, 0)	(0, 0, 1)	(0, 0, 0)	(0, 0, 0)	(0, 0, 0)	(0, 0, 0)	(0, 0, 0)

^a^Chi-square test, *p < 0.05.

^1^Maximum score of specific performance = 9; possible maximum score of Child-OIDP = 100; ^2^Percentiles (25, 50, 75).

Sample did not equal to N = 3285 due to missing data, i.e. 2 and 11 from intervention and control group respectively.

The overall prevalence of OIDP in the intervention group was lower at 57.8% compared to 60.8% in the control group. However the difference of 3.0% was not statistically significant. The prevalence of impact in each of the eight performances in the intervention group was consistently lower than those of the control group, respectively. The prevalence of oral impact on cleaning teeth was 28.8% in the intervention group versus 32.8% in the control group. The difference of 4.0% was statistically significant (*p* = 0.034).

In terms of OIDP score, the mean total score in the intervention group was 7.10. This score was lower than that in the control group (7.77). However, the difference of 0.67 was not statistically significant. Similar trend was also observed in each of the eight daily performances between the intervention and control group.

Table 3 shows levels of impact intensity of the eight performances of the Child-OIDP between the intervention and control group. In each of the eight performances, the majority in the intervention and control group had ‘very little’ to ‘moderate’ levels of impact intensity. Only small proportions in both groups reported having ‘severe’ and ‘very severe’ levels of impact intensity in all eight performances. At ‘very severe’ level of impact intensity, the proportion of intervention children implicated in each of the eight performances was consistently lower than that in the control group. The proportion of intervention children with ‘very little’ level of impact intensity on cleaning teeth (9.5%) was significantly higher than that in the control group (6.7%, pos hoc *p* = 0.002). Similarly, the proportion of intervention children with ‘little’ level of impact intensity on emotional stability (8.7%) was significant higher than that in the control group (6.2%, pos hoc *p* = 0.007). On the other hand, the proportion of intervention children with ‘very severe’ level of impact intensity on speaking (0.2%) was significantly lower than that in the control group (0.8%, pos hoc *p* = 0.009).

**Table 3:** **Weighted prevalence of intensity of impacts of the 8 performances of the Child**-**OIDP** (**n** = **3272**)

	**Performance**								
**Oral impacts**	**Eating (n = 1329)**	Speaking* (n = 642)	Cleaning* (n = 1037)	**Relaxing (n = 640)**	Emotion* (n = 799)	**Smiling (n = 745)**	**Study (n = 487)**	**Socialising (n = 630)**	**Total %**
Impact intensity % (all)	*40.2*	*19.3*	*31.3*	*19.2*	*24.1*	*22.5*	*14.5*	*18.8*	
Very Little	(*12.7*)	(*6.4*)	(*8.4*)	(*5.5*)	(*6.4*)	(*6.3*)	(*4.3*)	(*4.9*)	
Intervention	12.9	5.2	9.5^α^	5.2	5.5	6.5	4.0	4.5	50.5
Control	12.7	7.1	6.7^α^	5.7	6.9	6.2	4.5	5.2	57.8
Little	(*13.4*)	(*5.9*)	(*9.8*)	(*5.9*)	(*7.1*)	(*6.5*)	(*4.2*)	(*5.3*)	
Intervention	12.5	6.2	9.8	5.8	8.7^α^	5.7	4.3	5.2	58.2
Control	14.0	5.6	9.8	6.0	6.2^α^	7.0	4.1	5.3	58.0
Moderate	(*9.7*)	(*4.9*)	(*8.4*)	(*5.2*)	(*6.9*)	(*6.3*)	(*4.0*)	(*5.8*)	
Intervention	10.5	5.1	7.9	4.1	6.0	6.1	4.0	5.8	49.5
Control	9.2	4.8	8.6	5.9	7.5	6.4	4.0	5.8	52.2
Severe	(*3.6*)	(*1.6*)	(*3.5*)	(*1.9*)	(*2.3*)	(*2.1*)	(*1.3*)	(*1.8*)	
Intervention	3.5	1.8	3.5	2.3	2.0	2.3	1.2	2.0	18.6
Control	3.6	1.4	3.5	1.7	2.4	2.1	1.3	1.7	17.7
Very severe	(*0.7*)	(*0.6*)	(*1.2*)	(*0.7*)	(*1.4*)	(*1.3*)	(*0.8*)	(*1.0*)	
Intervention	0.2	0.2^α^	0.8	0.6	1.1	1.0	0.2	0.6	4.7
Control	1.0	0.8^α^	1.5	0.7	1.6	1.5	1.1	1.3	9.5

Intervention n = 1222; Control n = 2050.

*the difference between reports by children in the intervention and control group is statistically significant, *p < 0.05.

^α^Chi-square post hoc analysis with Bonferroni adjustment.

**Figure 1:**
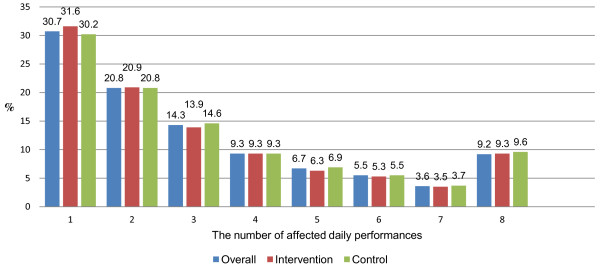
Weighted percentage distribution of the extent of impacts (number of OIDP performances affected by oral conditions) among individuals with impacts in the intervention and control group (n = 1971).

Overall, 75.7% and 74.9% of children in the intervention and control group had up to four performances affected by their oral conditions, respectively. The percentage of children in both groups who reported having five, six, and seven performances affected by their oral conditions were decreasing from less than 7% (five performances affected) to less than 4.0% (seven performances affected), respectively. Less than 10% of children in the intervention and control group reported having all eight daily performances affected by their oral conditions, respectively. The differences in the prevalence between the intervention and control group were not statistically significant (Figure 1).

In terms of perceived causes of oral impacts, the proportion of intervention children in each of the reported causes of oral impacts was consistently lower than that in the control group. The top five reported causes of oral impacts were toothache, fractured permanent tooth, sensitive tooth, bleeding gums and new tooth erupting in the intervention and control group, respectively. Presence of plaque/calculus, missing tooth and deformity of the mouth/face were the three least reported causes of oral impacts in both groups, respectively. The prevalence of intervention children who reported bleeding gums (31.4%) and presence of plaque or calculus (7.5%) as causes of oral impacts were significantly lower than that in the control group, respectively (35.5%, *p* = 0.016; 9.7%, *p* = 0.032) (Table 4).

**Table 4:** Weighted prevalence of perceived conditions causing oral impacts

**Perceived causes of oral impacts**	**Intervention (%)**	**Control (%)**
Toothache	46.7	48.9
Broken/fractured permanent tooth	43.0	45.4
Sensitive tooth	37.7	41.6
Bleeding gum*	31.4	35.5
New tooth erupting	34.2	34.2
Bad breath	30.0	30.3
Tooth decay/hole in tooth	28.6	31.5
Crowding/position of teeth	27.5	28.2
Exfoliating primary tooth/loose milk tooth	26.3	27.0
Swollen/inflamed gum	21.6	20.7
Colour of teeth/discoloured teeth	21.3	22.3
Shape or size of teeth	15.3	15.7
Oral ulcer	14.0	13.6
Spacing due to unerupted permanent tooth	8.2	8.7
Plaque or calculus*	7.5	9.7
Missing tooth	6.5	7.1
Deformity of mouth or face	2.0	3.2

Multiple responses were permitted.

Chi-square test, * *p* < 0.05.

## Discussion

The effectiveness of health promoting schools depends a lot on persuasive peer pressure created and endorsed by teacher trainers. In the DMP model, the programme provided the opportunities and platform for all health promotion activities in the school, including oral health. This arrangement lends social support, credibility and legitimacy for the comprehensive health promotion programme. It is well accepted because it is a natural extension of the health education programme of the Ministry of Health (MOH) to empower schools to create a healthy environment and maintain health awareness at a constant high level. Such a health-promoting environment cannot be sustained without the support of all stakeholders, i.e. DM’s, teachers, parents and MOH educators.

This present study was the first study conducted in Malaysia to evaluate the effect of health promoting schools on children’s OHRQoL. This study found that, overall, the DMP in Malaysian primary schools showed some benefits on 11–12 year olds’ OHRQoL after six years. This finding was comparable with a related study in Brazil where children who received a combination of dental treatment and oral health education in a primary care setting had a significantly better OHRQoL than children who received oral health education only [9]. The main difference between our study and that conducted in Brazil was our study did not include clinical oral prevention such as fissure sealants, fluoride therapy and simple fillings as part of the DMP. This was because in Malaysia, all primary schools had received these services free of charge as part of the Ministry of Health’s school dental services.

In our study, the overall prevalence of oral impacts was 59.7% (i.e. 57.8% and 60.8% in the intervention and control group, respectively). This prevalence was lower than past published studies on a similar age group in Thailand, Brazil, France and Italy [25-31], but higher than those in Tanzania and Sudan [32,33]. The most prevalent oral impacts were related to eating, followed by cleaning teeth and emotional stability while the least prevalent oral impacts were related to socialising and studying. A significant difference between the intervention and control group was seen in the prevalence of oral impact on cleaning teeth where the proportion of intervention children was significantly lower than that of control children.

The overall mean total impact score was 7.52. This score was lower than those in the Thailand studies [25,28], but higher than those in the France and Brazilian studies [26,29]. Similarly, the intervention group had lower overall and individual impact scores on the eight performances compared with those in the control group. However the differences were not statistically significant. The trend in favour of the intervention group could be attributed to the DMP which was carried out all year long in DMP schools compared to a short once-a-year dental treatment and oral health education programme in all schools by the School Dental Service.

The DMP health education component comprised peer-to-peer knowledge transfer and skill enhancement through a range of health related activities at school. Personal hygiene is one of the key areas and emphasis is placed on oral hygiene as well as other healthy practices such as proper hand-washing before meal, having short nails, clean clothes, well groomed hair and clean school areas. Over six years, the DMP gradually helped children to inculcate positive hygiene behaviours including regular tooth brushing and flossing at school as well as at home. Such efforts over time would be expected to contribute towards promoting children’s health and oral health at school. However, the evidence of effect on OHRQoL was equivocal. It may be that quality of life improvement may take longer to manifest. However, it was encouraging to note that children in the intervention group had a significantly lower prevalence of oral impacts related to cleaning teeth compared to children in the control group.

More than half of children with oral impacts in the intervention and control group reported ‘very little’ or ‘little’ levels of impact intensity in the eight daily performances, respectively. These findings were comparable to similar studies on the Thailand schoolchildren [25,34]. Only small proportions in both groups reported ‘severe’ or ‘very severe’ levels of impact intensity, respectively. The low levels of impact intensity reported in the study could be explained by the low levels of oral disease among Malaysian schoolchildren. According to the National Oral Health Survey of Schoolchildren in 2007, the 12-year-olds’ caries prevalence in Negri Sembilan was low at 32.8% while the mean DMFT and DMFS scores were 0.67 and 0.91, respectively [35]. Findings from the latest survey in 2012 had not been officially published yet. However, based on the current oral health trends, improvements in living standard, the fluoridation of water supply (0.4-0.6 mg/L with over 97% coverage) and a comprehensive free school dental service, the schoolchildren’s caries prevalence and score were expected to be even lower [18]. Thus, the low levels of impact intensity in children with oral impacts were probably due to low caries experience in this population. We anticipate if the disease levels were higher or more severe, the levels of impact intensity might be more severe.

Between the intervention and control group, the effect of DMP could be seen by the fact that significantly higher proportions of intervention children reported having ‘very little’ and ‘little’ levels of impact intensity on cleaning teeth and emotional stability, respectively. In addition, at a higher level of impact intensity, the number of intervention children with ‘very severe’ level of impact intensity on speaking was significantly lower than that of the control children.

In the present study, the most common cause of impacts was toothache, followed by fractured tooth and sensitive tooth in both groups, respectively. Between the groups, the significantly lower proportions of intervention children reported bleeding gums and presence of plaque or calculus as causes of oral impacts could be attributed to the weekly toothbrushing and flossing activities in DMP which led to significant improvements in children’s gingival health and levels of oral hygiene after six years as compared to children from non-DMP schools.

In terms of the extent of impacts, three in four children with impacts had 1–4 daily performances affected (out of eight performances). Although the prevalence was lower than that in a similar study in Thailand [25], the consequences on the children’s daily life and those of their family could be severe. Toothache, the most reported cause of impacts, may interfere with children’s daily routines leading to difficulty in eating, taking time off from school, inability to sleep at night and emotional disturbances. As children do not live in isolation, these impacts would also affect other family members. Also, less than one in ten children with impacts reported having all eight daily performances affected. These children were identified for further oral health assessment and treatment if necessary. This was done for ethical reasons because although the questionnaires were answered anonymously, the coded index numbers can be used to trace needy children if urgent referral and treatment were necessary. This was an example of how the Child-OIDP index could be used to identify high-risk individuals and prioritise treatment accordingly.

One of the limitations in the present study was that we had to assume there were no significant difference in children’s OHRQoL at baseline (in 2006) between the intervention and control group. Another limitation that might influence the finding was related to the possibility of selection bias where there were more children from rural schools in the control group as compared to those in the intervention group. However, this limitation was overcome by using weighted data in the analysis. A few illiterate students had to be excluded from the sample because they were not able to read or understand the questionnaire. However, their number was very small (n = 44, 1.2%) to have a significant influence on the overall results. A few students needed help in understanding the instructions. To overcome this, the researcher gave additional verbal explanation before the questionnaire was answered. Additionally, two teacher facilitators from each school were trained to help the students only when necessary. We suggest future studies might consider using validated interview-based questionnaire if it involved illiterate populations but this will entail more logistics problems.

Another limitation was that with any self-report, the perception of children might be influenced by the way they looked after their teeth. For example, if they had brushed regularly, they might tend to feel better about their teeth and perceived to have good oral health regardless of the actual state of their mouth and teeth.

Further research should consider evaluating the DMP effect on children’s general health and related parameters. Process evaluation to evaluate how well the programme has been implemented and accepted by everyone is also recommended. This should involve key stakeholders and the students. Evidence of impact on children’s health and well being, the school environment and academic achievement should be comprehensively evaluated to determine the long term effects of health promoting schools.

## Conclusions

Children from health-promoting schools (DMP) had significantly better OHRQoL compared to children from non-DMP schools where the DMP children had less episodes of oral impact on cleaning teeth, lower proportion with ‘very severe’ level of impact intensity on speaking, and higher proportion with ‘very little’ and ‘little’ levels of impact intensity on cleaning teeth and emotional stability respectively. DMP children also reported less episodes of bleeding gum and presence of calculus/plaque as the cause of oral impacts.

## Abbreviations

Child-OIDP: Child oral impacts on daily performance; OHRQoL: Oral health related quality of life; DMP: *Doktor Muda* programme; DM: *Doktor Muda.*

## Competing interests

The authors declare that they have no competing interests.

## Authors’ contributions

ZYMY contributed in the study design, acquisition of data, analysis and interpretation of data, and drafting the manuscript. NJ advised on the study design, data collection, data analysis and editing the manuscript. Both authors read and approved the final manuscript.
